# Cost effectiveness of intracameral cefuroxime prophylaxis and its efficacy in preventing endophthalmitis after cataract surgery in a referral hospital

**DOI:** 10.5935/0004-2749.20230052

**Published:** 2023

**Authors:** Lívia da Silva Conci, Arthur Pinheiro Favarato, Alexandre Grobberio Pinheiro

**Affiliations:** 1 Department of Ophthalmology, Universidade Federal do Espirito Santo, Vitória, Espírito Santo, Brazil.; 2 Department of Ophthalmology, Universidade Estadual de Campinas, Campinas, São Paulo, Brazil.

**Keywords:** Cataract extraction, Endophthalmitis, Cefuroxime, Antibiotic prophylaxis, Cost-effectiveness evaluation, Extração de catarata, Endofitalmite, Cefuroxima, Antibioticoprofilaxia, Avaliação de custo-efetividade

## Abstract

**Purpose:**

To present the results of a retrospective study regarding the clinical and economic impact of intracameral cefuroxime administration to prevent endophthalmitis during cataract surgery in a referral hospital.

**Methods:**

This study included 16,902 eyes from patients who had undergone cataract surgery between 2013 and 2017. From May 2014 onwards, all patients received routine intracameral injections of 1 mg cefuroxime (10 mg/1 mL) after phacoemulsification. The prophylactic efficacy was evaluated using the relative risk ratio, whereas the economic impact was evaluated using number needed to treat to avoid endophthalmitis.

**Results:**

Before introducing cefuroxime, 3,407 cataract surgeries were performed using the phacoemulsification technique, and 7 post-operatory cases of endophthalmitis occurred (0.2% incidence). After introducing the cefuroxime protocol, 13,495 surgeries were performed, and 4 endophthalmitis cases were registered (0.03% incidence). Cefuroxime was identified as a protective factor against the development of endophthalmitis [risk ratio = 14%, p=0.002, 95% confidence interval (CI) 95%, 4%-49%], with an economic impact of number needed to treat = 568. The potential savings with cefuroxime was approximately US $2,334.36 for every 568 patients treated.

**Conclusion:**

The incidence of endophthalmitis decreased by 86% (risk ratio = 14%, p=0.002, 95% CI, 4%-49%) after introducing intracameral cefuroxime prophylaxis at the study hospital. The results presented herein provide strong evidence for the use of cefuroxime in endophthalmitis prophylaxis after phacoemulsification surgeries, outperforming the alter­native by providing both economic and clinical benefits.

## Abstract (pt)

**Objetivo:**

Apresentar os resultados de um estudo retrospectivo sobre o impacto clínico e econômico da administração de cefuroxima intracameral para prevenir endoftalmite nas cirurgias de catarata em um hospital de referência.

**Métodos:**

Este estudo incluiu 16.902 olhos de pacientes submetidos à cirurgia de catarata entre 2013 e 2017. A partir de maio de 2014, todos os pacientes receberam rotineiramente uma injeção intracameral de 1mg de cefuroxima (10mg/1mL) ao final da cirurgia de facoemulsificação. A eficácia da profilaxia foi avaliada usando o risco relativo e o impacto econômico foi avaliado com o número necessário para tratar para se evitar um caso de endoftalmite.

**Resultados:**

Antes da introdução do protocolo da cefuroxima, foram realizadas 3.407 cirurgias de catarata por facoemulsificação e ocorreram 7 casos de endoftalmite pós-operatória (incidência de 0,2%). Após a introdução do protocolo da cefuroxima, foram realizadas 13.495 cirurgias e registrados 4 casos de endoftalmite (incidência de 0,03%). A cefuroxima foi um fator de proteção no desenvolvimento de endoftalmite (risco relativo = 14%, p=0,002, Intervalo de Confiança de 95% [IC 95%], 4% - 49%) e o impacto econômico do número necessário para tratar = 568. A economia potencial com a cefuroxima foi de aproximadamente US$ 2.334,36 para cada 568 pacientes tratados.

**Conclusão:**

A incidência de endoftalmite diminuiu 86% (risco relativo = 14%, p=0,002, IC 95% 4% - 49%) desde a introdução da profilaxia com cefuroxima intracameral no hospital do estudo. Os resultados apresentados mostram forte evidência para o uso da cefuroxima na profilaxia da endoftalmite após cirurgias de facoemulsificação, por proporcionar economia de custos e benefício clínico.

## INTRODUCTION

Although infrequent, acute endophthalmitis is one of the most dreaded postoperative complications following cataract surgery, potentially leading to unfavorable visual outcomes, with 15%-30% of affected cases developing a visual acuity worse or equivalent to 20/200^(1-3)^. The incidence of this complication varies according to region. For instance, a 2010 study in Brazil reported an incidence rate of 0.3%^(4)^, whereas a 2013 study in Sweden revealed an incidence rate of 0.03%^(5)^.

Measures for preventing surgical site infections can be divided into actions taken in the pre-, intra-, and postoperative stages. In this context, the topical use of preoperative iodopovidone has already been a well-established and widely adopted surgical prophylaxis^(6)^. Regarding the prophylactic use of antibiotics, the European Society of Cataract and Refractive Surgeons (ESCRS), following evidence provided by a Swedish study^(7)^, conducted the first multicenter randomized clinical trial that demonstrated the efficacy of including perioperative intracameral cefuroxime injections with other previously existing measures, with patients not receiving cefuroxime being approximately five times more likely to develop endophthalmitis: odds ratio, 4.92, 95% CI 1.87-12.9^(8)^. The aforementioned study, together with others presenting similar outcomes^(9-13)^, served as the basis for implementing this prophylaxis in the referral ophthalmologic hospital studied herein. In line with this, Rodriguez-Caravaca et al. demonstrated that the prophylactic use of cefuroxime not only reduced the incidence of endophthalmitis but also provided positive economic benefits^(12)^. Afterwards, similar results were seen again in studies from different countries^(14-16)^.

The present study was conducted following the safe practices for the dilution and use of intracameral cefuroxime obtained from intravenous preparations implemented since May 2014. Moreover, this study is the first Brazilian study to report on the large-scale use of intracameral cefuroxime for endophthalmitis prophylaxis after phacoemulsification surgeries. The main objective was to retrospectively determine whether the introduction of this prophylaxis would reduce the incidence rates of endophthalmitis and evaluate its economic impact.

## METHODS

### Study population

The present study included patients at Hospital Capixaba de Olhos, located in Vitória, Espírito Santo, Brazil, a recognized referral hospital for ophthalmological cases.

All patients undergoing phacoemulsification surgery from May 2013 to December 2017 were identified by electronically searching the medical records in the DATASIGH system based on requests for phacoemulsification surgery. Subsequently, procedures combined with phacoemulsification, such as phacoemulsification associated with vitrectomy or trabeculectomy, were excluded.

### Determination of cases

After surgery, all patients routinely received clear instructions to urgently seek ophthalmologic care in the presence of symptoms suggestive of postoperative endophthalmitis, such as ocular pain or worsening of visual acuity. The Hospital Infection Control Committee (HICC) actively searched for cases by inquiring for the presence of such symptoms via telephone, which was performed from the first week after surgery in all cases.

Postoperative patients with pain, hypopyon, hazy anterior chamber, vitreitis or vision loss attributed to infection, with no other identified cause of intraocular inflammation (such as Toxic Anterior Segment Syndrome or uveitis) were diagnosed with presumed bacterial endophthalmitis and were referred to the HICC. Following current recommendations^(17)^, such cases must undergo collection of intraocular fluid samples for microbiological analysis and intravitreal injections of ceftazidime and vancomycin, in addition to performing posterior vitrectomy via pars plana, when indicated.

The present study included all presumed endophthalmitis cases after phacoemulsification surgery in the period from 01/05/2013 to 12/31/2017. Notably, no change in case detection protocol was observed throughout the entire study period. Moreover, the HICC has a strict protocol for detecting and treating any presumed endophthalmitis.

### Antibiotic prophylaxis protocol

The reference hospital has a uniform protocol for cataract surgery, which is adopted by all surgeons. Since its inauguration in March 2006, patients at this hospital have been routinely prepared for surgery using the 5% povidone-iodine solution as a topical antiseptic agent. The procedures are performed with topical anesthesia or peribulbar block. In the postoperative period, patients used antibiotic (4th generation quinolones) and corticosteroids, with or without non-steroidal anti-inflammatory drugs. Since May 2014, following the guidelines of the ESCRS study^(8)^, the protocol has been updated, with cefuroxime approved for intraocular use by the HICC. Prior to this date, no surgeon used intracameral injection, and after this protocol modification, all patients undergoing cataract surgery routinely received prophylactic intracameral cefuroxime (1 mg/0.1 mL) at the end of surgery. Antisepsis with 5% iodine-povidone and regular eye drops prescriptions were maintained postoperatively.

As intracameral cefuroxime (Aprokam^®^) is not commercially available in Brazil, the intracameral solution is obtained from the dilution of an intravenous preparation of cefuroxime (Zinacef^®^) in balanced saline solution (BSS), inside the operating room, maintaining the rules of good practices.

The protocol follows the steps:

1. 1 vial/750 mg ampoule + 7.5ml BSS = 100mg/ml solution
2. 1mL of 100mg/mL solution + 9mL of BSS = 10mg/mL ready solution
3. Aspirate 0.2mL and inject 0.1mL in the anterior chamber at the end of surgery.

### Cost evaluation

Hospital costs for a dose of cefuroxime and for a case of endophthalmitis were calculated using full-cost analysis, considering all hospital expenses, which include medications, syringes, tubing, surgical kits, and surgeon time.

The calculation of the cefuroxime prophylaxis considered the average prices of Zinacef^®^ ampoules, syringes, and needles throughout the period. One ampoule of Zinacef^®^ was diluted before the start of the surgeries for all patients scheduled for surgery on that day. The remainder of the solution obtained was kept at the end of the day if not used. No more than one vial of Zinacef^®^ was needed per day of surgery. The price of BSS was considered negligible considering that it was removed from part of the BSS bag of the first surgery. Thereafter, the total number of days that surgery occurred during the study period was calculated, thereby obtaining the total number of vials of Zinacef^®^ utilized, which was multiplied by the average price of this medication in this period. This value was then added to the average value of needles and syringes used for all patients included herein, subsequently divided by the total number of patients, obtaining the mean value for prophylaxis for each patient.

### Comparison groups

The main objective of this study was to compare the incidence of presumed infectious endophthalmitis between patients who did not receive intracameral cefuroxime (Group 1-May 1^st^, 2013 to May 25^th^, 2014) and those in whom intracameral cefuroxime was admi­nistered (Group 2-May 26, 2014 through December 31^st^, 2017). The incidence rate of endophthalmitis was calculated from the relationship between the number of cases of presumed endophthalmitis identified in the period and the number of phacoemulsification surgeries performed in the period multiplied by 100. The incidence of endophthalmitis before the use of cefuroxime was also analyzed using the national incidence rates of endophthalmitis in the literature.

### Data analysis

The efficacy of cefuroxime was assessed using relative risk (RR), with statistical significance set at a p value of <0.05 calculated using Fisher’s exact test. The number needed to treat (NNT) assessed the impact of prophylaxis use. For comparison between studies, the chi-square test was used. All analyses were performed using SPSS 20.0.

## RESULTS

This study included 16,902 phacoemulsification surgeries performed by 81 surgeons. Based on the records, 3,407 patients from Group 1 (05/01/2013 to 05/25/2014) and 13,495 patients from Group 2 (05/26/2014 to 12/31/2017) participated in the study. From 2013 to 2017, 11 cases of endophthalmitis were reported at the hospital. The frequency of presumed infectious endophthalmitis throughout the study period was 0.065%. The incidence of endophthalmitis varied every year, with values fluctuating between 0.15% and 0.03% (Table 1).

**Table 1:** Presumed endophthalmitis incidence by year

Year	Incidence (%)
2013	2/2.202 (0.09)
2014	5/3.438 (0.15)
2015	1/3.707 (0.03)
2016	1/3.686 (0.03)
2017	2/3.869 (0.05)

Table 2 shows some characteristics of the patients with endophthalmitis. The average age was 68.18 years old (58 to 80 years), while the average number of days between surgery and diagnosis was 10.27 days.

**Table 2:** Microbiological data and required treatment

Cases of presumed endophthalmitis	Date of surgery	Date of presentation (Days After Surgery)	Collection of material for analysis	Microbiological analysis results	Required treatment
1	07/23/2013	7	No		Intravitreal Injection
2	12/11/2013	2	No		Intravitreal Injection
3	03/20/2014	5	Yes	Negative	Intravitreal Injection
4	03/20/2014	56	No		Intravitreal injection + Vitrectomy
5	05/15/2014	2	Yes	*Pseudomonas aeruginosa*	Intravitreal injection + Vitrectomy
6	05/15/2014	12	Yes	Gram positive cocci in pairs. Negative Culture	Intravitreal injection + Vitrectomy
7	05/20/2014	2	Yes	*Morganella morganii*	Intravitreal injection + Vitrectomy
8	08/17/2015	10	Yes	Negative	Intravitreal injection + Vitrectomy
9	05/10/2016	3	Yes	*Staphylococcus haemolyticus*	Intravitreal injection + Vitrectomy
10	03/30/2017	8	Yes	Negative	Intravitreal injection
11	05/16/2017	6	Yes	Negative	Intravitreal injection + Vitrectomy

Between 05/01/2013 and 05/25/2014, 7 cases of en­do­phthalmitis were recorded in Group 1, corresponding to an incidence rate of 0.2%. From 05/26/2014 to 12/31/2017, 4 cases of endophthalmitis were reported in Group 2, corresponding to an incidence rate of 0.03%. The incidence of endophthalmitis was significantly higher in the group that did not receive intracameral cefuroxime, which was identified as a protective factor for the development of endophthalmitis (RR=0.14, p=0.002, CI 95%, 0.04-0.49). The impact or number of patients NNT to avoid an additional infection was 568. The average cost of one dose of cefuroxime per patient was US $0.16, whereas the average cost of treatment of a case with endophthalmitis, considering the cost of intravitreal injection and vitrectomy, was US $2,429. Out of 11 cases, in 7 required vitrectomies in combination with intravitreal injection. The potential savings with cefuroxime was approximately US $2,334.36 for every 568 patients treated.

## DISCUSSION

Endophthalmitis has been considered the most challenging complication following cataract surgery due to its poor outcomes and the potential for severe loss of visual function. Evaluating its incidence and preventive methods is a key aspect for preventing this disease entity. In this context, a meta-analysis compared the efficacy and safety of intracameral injections of cefuroxime, moxifloxacin, and vancomycin, subsequently demonstrating that both moxifloxacin and cefuroxime reduced the incidence of endophthalmitis, with minimal to no adverse events^(18)^. It is worth mentioning that the literature demonstrating a reduction in the risk of endophthalmitis with intracameral moxifloxacin were mostly retrospective studies^(19-22)^, with only one recent single-site randomized controlled clinical trial showing similar findings^(23)^. The intracameral use of vancomycin is not recommended given its association with hemorrhagic occlusive retinal vasculitis^(24)^.

In countries such as the United States and Brazil, intracameral cefuroxime (Aprokam^®^) is not commercially available. Therefore, the intracameral solution is obtained from the dilution of an intravenous preparation of cefuroxime in balanced saline solution in the operating room, which limits its large-scale use in these countries given that the need to prepare and dilute them in the operating room may facilitate dosing errors^(25-28)^. Once there is no Food and Drug Administration-approved product available for intracameral therapy, routine use of intracameral antibiotics should be carefully considered, and providers need to weigh the risk and benefits of therapy^(29)^.

The present study showed that between 2013 and 2017, the overall incidence of presumed endophthalmitis was 0.065%, with a higher rate (0.2%) in Group 1 (May 1, 2013 to May 25, 2014). Before prophylaxis was instituted, no significant difference was observed between our findings and those from another Brazilian study (0.3%, p=0.35)^(5)^. This changed after the adoption of prophylaxis from May 26, 2014 to December 31, 2017, reaching a 0.03% incidence in Group 2. Thus, the introduction of intracameral injections resulted in an 86% reduction in the rate of presumed endophthalmitis in the studied hospital. These data corroborate the efficacy of cefuroxime in the prevention of endophthalmitis and are consistent with the results of the multicenter randomized clinical trial ESCRS, which proved the efficacy of perioperative intracameral cefuroxime injections, which promoted a five-fold reduction in the risk of endophthalmitis^(8)^.

The data presented herein provide further evidence that cefuroxime was a protective factor (RR=0.14, p=0.002, CI 95% 0.04-0.49). Had Groups 1 and 2 exhibit the same incidence rate, 27 new cases of endophthalmitis should have been observed within the study period. Thus, the introduction of the intracameral cefuroxime prophylaxis theoretically prevented 23 new cases. This reduction in incidence led to the prevention of approximately one case of endophthalmitis for every 568 patients treated with cefuroxime (NNT), with consequent savings of approximately US $2,334.36 for every 568 patients who received prophylactic cefuroxime. Rodriguez-Caravaca et al. also demonstrated the positive economic impact for cefuroxime prophylaxis, which was even greater than that suggested herein, with a potential saving of €1177 for every 182 patients trea­ted with prophylactic cefuroxime^(12)^. In the context of public health, these savings can have considerable social impact given that they allow investment in other fields and even increased number of procedures. Moreover, the current pandemic may require careful allocation of public health care funds. It should also be considered that such prophylaxis avoided irreparable damages, such as emotional factors, irreversible visual losses, and out-of-hospital expenses (removal from employment, transportation, and budget burden) despite not having been accounted in our study. Therefore, the cost savings suggested herein may underestimate the actual value given that it does not count for all of the patient’s expenses associated with treatment.

Our study has several limitations worth noting. One was its retrospective design, which may not the best approach for determining efficacy. Moreover, we could not obtain exact clinical information regarding endophthalmitis cases, such as visual acuity at presentation and following treatment. Another limitation was microbiological analysis given that we had three suspected cases in whom no microbiological sample was collected and four suspected cases with negative culture. Therefore, our culture positivity rate (4/11 = 36.36%) was lower compare to other studies^(7-10,12)^.

Despite these limitations, the present study in a Brazilian population suggests that the intracameral administration of cefuroxime was significantly cost effective and efficient. Our study corroborates the clinical trial results in a large clinical practice outcomes database, further confirming the value of the prophylactic approach in another parts of the world.

## References

[1] Results of the Endophthalmitis Vitrectomy Study (1995). A randomized trial of immediate vitrectomy and of intravenous
antibiotics for the treatment of postoperative bacterial endophthalmitis.
Endophthalmitis Vitrectomy Study Group. Arch Ophthalmol.

[2] Kamalarajah S, Silvestri G, Sharma N, Khan A, Foot B, Ling R (2004). Surveillance of endophthalmitis following cataract surgery in the
UK.”. Eye (Lond).

[3] Kernt M, Kampik A (2010). Endophthalmitis: pathogenesis, clinical presentation, management,
and perspectives. Clin Ophthalmol.

[4] Melo GB, Bispo PJ, Regatieri CV, Yu MC, Pignatari AC, Höfling-Lima AL (2010). Incidence of endophthalmitis after cataract surgery (2002-2008)
at a Brazilian university-hospital. Arq Bras Oftalmol.

[5] Friling E, Lundström M, Stenevi U, Montan P (2013). Six-year incidence of endophthalmitis after cataract surgery:
Swedish national study. J Cataract Refract Surg.

[6] Speaker MG, Menikoff JA (1991). Prophylaxis of endophthalmitis with topical
povidone-iodine. Ophthalmology.

[7] Montan PG, Wejde, Koranyi G, Rylander M (2002). Prophylactic intracameral cefuroxime: efficacy in preventing
endophthalmitis after cataract surgery. J Cataract Refract Surg.

[8] (2007). Prophylaxis of postoperative endophthalmitis following cataract
surgery: results of the ESCRS multicenter study and identification of risk
factors. J Cataract Refract Surg.

[9] Shorstein NH, Winthrop KL, Herrinton LJ (2013). Decreased postoperative endophthalmitis rate after institution of
intracameral antibiotics in a Nothern California eye
department. J Cataract Refract Surg.

[10] García-Sáenz MC, Arias-Puente A, Rodriguez-Caravaca G, Bañuelos JB (2010). Effectiveness of intracameral cefuroxime in preventing
endophthalmitis after cataract surgery: Ten-year comparative
study. J Cataract Refract Surg.

[11] Barreau G, Mounier M, Marin B, Adenis JP, Robert PY (2012). Intracameral cefuroxime injection at the end of cataract surgery
to reduce the incidence of endophthalmitis: French study. J Cataract Refract Surg.

[12] Rodriguez-Caravaca G, Garcia-Sáenz MC, Villar-Del-Campo MC, Andrés-Alba Y, Arias-Puente A (2013). Incidence of endophthalmitis and impact of prophylaxis with
cefuroxime on cataract surgery. J Cataract Refract Surg.

[13] Myneni J, Desai SP, Jayamanne DG (2013). Reduction in postoperative endophthalmitis with intracameral
cefuroxime. J Hosp Infect.

[14] Rahman N, Murphy CC (2015). Impact of intracameral cefuroxime on the incidence of
postoperative endophthalmitis following cataract surgery in
Ireland. Ir J Med Sci.

[15] Katz G, Blum S, Leeva O, Axer-Siegel R, Moisseiev J, Tesler G (2015). Intracameral cefuroxime and the incidence of post-cataract
endophthalmitis: an Israeli experience. Graefes Arch Clin Exp Ophthalmol.

[16] Beselga D, Campos A, Castro M, Fernandes C, Carvalheira F, Campos S (2014). Postcataract surgery endophthalmitis after introduction of the
ESCRS protocol: a 5-year study. Eur J Ophthalmol.

[17] Lemley CA, Han DP (2007). Endophthalmitis: a review of current evaluation and
management. Retina.

[18] Bowen RC, Zhou AX, Bondalapati S, Lawyer TW, Snow KB, Evans PR (2018). Comparative analysis of the safety and efficacy of intracameral
cefuroxime, moxifloxacin and vancomycin at the end of cataract surgery: a
meta-analysis. Br J Ophthalmol.

[19] Matsuura K, Miyoshi T, Suto C, Akura J, Inoue Y (2013). Efficacy and safety of prophylactic intracameral moxifloxacin
injection in Japan. J Cataract Surg.

[20] Haripriya A, Chang DF, Namburar S, Smita A, Ravindran RD (2016). Efficacy of intracameral moxifloxacin endophthalmitis prophylaxis
at Aravind Eye Hospital. Ophthalmology.

[21] Vieira IV, Boianovsky C, Saraiva TJ, Godoy RB, Lake J (2017). Safety and efficacy of intracameral moxifloxacin injection for
prophylaxis of endophthalmitis after phacoemulsification. Arq Bras Oftalmol.

[22] Haripriya A, Chang DF, Ravindran RD (2017). Endophthalmitis reduction with intracameral moxifloxacin
prophylaxis: analysis of 600 000 surgeries. Ophthalmology.

[23] Melega MV, Alves M, Cavalcanti Lira RP, Cardoso da Silva I, Ferreira BG, Assis Filho HL (2019). Safety and efficacy of intracameral moxifloxacin for prevention
of post-cataract endophthalmitis: Randomized controlled clinical
trial. J Cataract Refract Surg.

[24] Witkin AJ, Chang DF, Jumper JM, Charles S, Eliott D, Hoffman RS (2017). Vancomycin-associated hemorrhagic occlusive retinal vasculitis:
clinical characteristics of 36 eyes. Ophthalmology.

[25] Buyukyildiz HZ, Gulkilik G, Kumcuoglu YZ (2010). Early serous macular detachment after phacoemulsification
surgery. J Cataract Refract Surg.

[26] Sakarya Y, Sakarya R (2010). Cefuroxime dilution error. Eur J Ophthalmol.

[27] Olavi P (2012). Ocular toxicity in cataract surgery because of inaccurate
preparation and erroneous use of 50mg/ml intracameral
cefuroxime. Acta Ophthalmol.

[28] Delyfer MN, Rougier MB, Leoni S, Zhang Q, Dalbon F, Colin J (2011). Ocular toxicity after intracameral injection of very high doses
of cefuroxime during cataract surgery. J Cataract Refract Surg.

[29] George NK, Stewart MW (2018). The routine use of intracameral antibiotics to prevent
endophthalmitis after cataract surgery: how good is the
evidence?. Ophthalmol Ther.

